# Gender-Specific Metabolomics Approach to Kidney Cancer

**DOI:** 10.3390/metabo11110767

**Published:** 2021-11-10

**Authors:** Stanisław Deja, Adam Litarski, Karolina Anna Mielko, Natalia Pudełko-Malik, Wojciech Wojtowicz, Adam Zabek, Tomasz Szydełko, Piotr Młynarz

**Affiliations:** 1 Center for Human Nutrition, The University of Texas Southwestern Medical Center, Dallas, TX 75390, USA; 2 Department of Biochemistry, The University of Texas Southwestern Medical Center, Dallas, TX 75390, USA; 3 Faculty of Chemistry, Opole University, Pl. Kopernika 11a, 45-040 Opole, Poland; 4 Department of Urology, 4th Military Clinical Hospital, 50-981 Wroclaw, Poland; adam.litarski@gmail.com; 5 Department of Biochemistry, Molecular Biology and Biotechnology, Faculty of Chemistry, Wroclaw University of Science and Technology, Wybrzeże Wyspiańskiego 27, 50-370 Wrocław, Poland; karolina.mielko@pwr.edu.pl (K.A.M.); natalia.pudelko-malik@pwr.edu.pl (N.P.-M.); wojciech.wojtowicz@pwr.edu.pl (W.W.); adam.zabek@innobiotix.com (A.Z.); 6 University Center of Excellence in Urology, Wroclaw Medical University, 50-556 Wroclaw, Poland; tomasz.szydelko1@gmail.com

**Keywords:** renal cell carcinoma, RCC, metabolomics, serum, NMR, gender, male, female

## Abstract

Renal cell carcinoma (RCC) is the most common form of kidney malignancy. RCC is more common among men with a 2/1 male/female incidence ratio worldwide. Given the underlying epidemiological differences in the RCC incidence between males and females, we explored the gender specific ^1^H NMR serum metabolic profiles of RCC patients and their matched controls. A number of differential metabolites were shared by male and female RCC patients. These RCC specific changes included lower lactate, threonine, histidine, and choline levels together with increased levels of pyruvate, *N*-acetylated glycoproteins, beta-hydroxybutyrate, acetoacetate, and lysine. Additionally, serum lactate/pyruvate ratio was a strong predictor of RCC status regardless of gender. Although only moderate changes in metabolic profiles were observed between control males and females there were substantial gender related differences among RCC patients. Gender specific metabolic features associated with RCC status were identified suggesting that different metabolic panels could be leveraged for a more precise diagnostic.

## 1. Introduction

The renal cell carcinoma (RCC) is the most common form of kidney malignancy constituting more than 90% of all kidney cancer cases [1]. The most frequently occurring forms of RCC are clear cell renal cancer carcinoma (ccRCC), papillary renal cancer cell carcinoma (pRCC), and chromophobe cancer cell carcinoma (crRCC) [1]. The established risk factors for RCC include genetic background, cigarette smoking, obesity, hypertension, and acquired cystic kidney disease [2,3]. The incidence of kidney cancer shows a relationship with the degree of country or region development, with the highest incidence rates being recorded in North America, Western Europe, and Australia, while South America, Africa, and Asia display relatively low number of cases [4,5,6]. Additionally, gender was reported to influence incidence, histology, and response to medical therapy in RCC as well [7].

Kidney metabolism may differ between men and women, as many gender related differences in renal function and resulting blood pressure have been observed in animals and in humans [8,9]. Furthermore, RCC has a consistent 2/1 (male/female) incidence ratio worldwide [10,11,12], with the lowest variability of this ratio comparing to other solid tumors [13]. While differences in cancer incidence between men and women are commonly attributed to different exposure to risk factors, this does not explain the observed 2/1 sex ratio for kidney cancer, which remains unchanged regardless of age, time period, and geographical region [13]. Men were reported to have greater risk of developing RCC than women [14] and tumors in men tend to be larger [15]. Moreover, men usually have more advance disease at the time of first diagnosis, while in case of metastatic RCC women seem to have a worse response to therapy and shorter survival time [7]. Currently, the gender related differences observed in RCC are incompletely understood. Particularly, an evaluation of the degree to which alterations in metabolic pathways contribute to gender related variability in RCC remains to be elucidated.

The current standard for RCC diagnosis is based on abdominal ultrasound scanning, MRI, CT PET, and intravenous pyelogram [16], while the invasive diagnostic methods include angiography and the fine needle biopsy [17]. However, currently there are no simple and cheap laboratory tests that can be used for the rapid diagnosis of early stage RCC and thus enable early treatment. Metabolomics is a comprehensive analytical approach for monitoring metabolite changes in biofluids and tissues, which over last two decades was progressing toward becoming a clinically useful tool for cancer diagnosis. It enables molecular studies for biomarker discovery and has potential to be used as a tool of personalized medicine allowing monitoring of disease progression or treatment [18].

A number of excellent metabolomics studies on RCC have already been conducted. These investigations were performed with the use of various analytical platforms such as NMR and MS using blood [19,20,21,22], urine [23,24], tissue samples [25,26], or a combination of different types of samples [27,28,29], and they indicate that metabolomics not only can enable RCC diagnosis but also could be useful for staging of the disease and selection of therapeutic targets. However, while very valuable for general RCC biomarker discovery, these studies rarely focused on gender related differences in metabolic profiles associated with RCC. Few reports have thus far investigated gender related metabolic profiles in RCC; however, even in these studies male and female groups were not always balanced resulting in ambiguous interpretations. Given the existence of differences in RCC incidents, prognosis, and molecular characteristics of some kidney tumors in this study we undertook an effort to quantify serum metabolic profiles of RCC patients and specifically evaluate the common and differential metabolites between male and female RCC patients. 

## 2. Results

### 2.1. Serum ^1^H NMR Profiles of RCC Patients

Across all 100 serum samples analyzed using ^1^H NMR, 44 metabolite resonances were consistently detected and showed sufficient signal to noise and resolution to be quantified (Table S1). Among these resonances, lipid signals were analyzed by means of chemical group. Two small molecule resonances remained unidentified: Unk1 (1.399 ppm) and Unk2 (1.424 ppm), while still being quantitatively analyzed. Initial data quality evaluation consisted of an unsupervised analysis, and Grubbs test which detected one sample with characteristics of a substantial outlier (Figure S1A). Upon closer evaluation, this sample was identified as a male control subject and was subsequently excluded due to a 3-fold upregulated lipid signals comparing to the average in control group (Figure S1B). At this stage a total of 99 samples, (control 26 males and 23 females; RCC 30 males and 20 females) were subjected to appropriate chemometric and univariate statistical analysis.

### 2.2. Discriminatory Potential of Serum in RCC Diagnosis Independent of Gender

**Figure 1:**
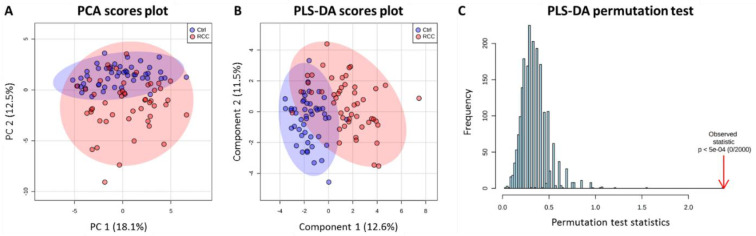
Chemometric analysis based on metabolites detected in serum of Control and RCC patients. (**A**) PCA scores plot, (**B**) PLS-DA scores plot, and (**C**) permutation test with respect to separation distance (2000 permutations).

First, we analyzed male and female patients jointly to identify gender independent metabolic features and their relation to RCC status. When the 99 samples were subjected to the unsupervised PCA analysis, the dataset showed moderate separation between the Control and RCC groups while maintaining partial overlap (Figure 1A). A more detailed analysis of different combinations of principal components indicated that separation was mostly associated with the first two dimensions of PCA, suggesting the existence of partial, although not overwhelming, metabolic differences between RCC patients and Controls. In order to evaluate the ability of ^1^H NMR serum metabolic profiles to discriminate between Control group and RCC patients a supervised analysis was conducted. PLS-DA model with satisfactory cross validation parameters (R^2^ = 0.61 and Q^2^ = 0.47) resulted in a substantial separation between groups (Figure 1B) and was highly statistically significant based on permutation test (*p* < 0.0005) (Figure 1C). Yet, still scores plot was characterized by some degree of overlap between the groups indicating high degree of variability particularly across RCC patients (Figure 1B).

The ability of individual metabolite signals to differentiate between groups was evaluated based on their Variable Importance Projections (VIPs) scores. Metabolites characterized by high VIP values were additionally subjected to single logistic regression (SLR) analysis, in order to evaluate their individual predictive power regarding RCC status. All metabolites with VIP score > 1 from PLS-DA and *p* value < 0.01 from logistic regression are reported in Table 1 along with their respective AUC, *p*-value, and percentage change. No lipids were found in this list and neither creatinine nor creatine were statistically different between the RCC and Control groups, which was somewhat unexpected given the fact that creatinine is a marker of renal function. Among the most significantly changed metabolites we found lactate, *N*-acetylated compounds (NAC2 δ = 2.05 ppm), threonine, and histidine, followed by ketones and choline (Table 1). 

**Figure 2:**
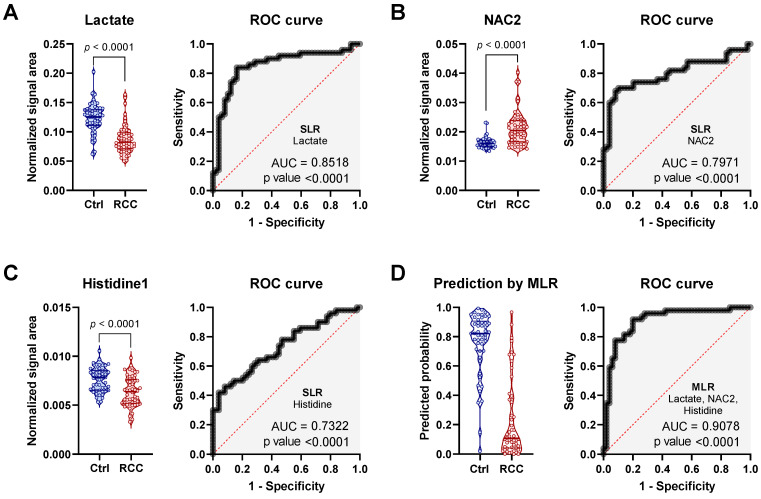
Statistical and predictive performance of three most differential metabolites. Normalized signal area ROC curve from SLM for (**A**) lactate, (**B**) *N*-acetylated compounds (NAC2 δ = 2.05 ppm), and (**C**) histidine. (**D**) Combined predictive power of lactate, NAC2 and histidine using MLR.

The top three most important metabolites except threonine are presented in detail in Figure 2. Threonine was not considered for further analysis, due to its very strong correlation with lactate, which is most likely caused by substantial degree of signal overlap between these two metabolites (data not shown). Based on the SLR results, a decrease in serum lactate was the strongest single metabolite variable predictor of RCC status and showed a robust ROC curve with AUC of 0.85 (Figure 2A). An increase in NAC2 was characterized by a good ROC performance and AUC of 0.80 (Figure 2B), while a decrease in histidine, even though significant, was characterized by moderate ROC performance with AUC of 0.73 (Figure 2C). Importantly, the combination of these three most differential metabolites using multiple logistic regression (MLR) model generated superior performance with AUC of 0.91 and positive predictive power of 89% (Figure 2D).

Interestingly, metabolite signals associated with ketone body metabolism were also detected as significantly increased in serum of RCC patients comparing to Controls (Table 1). This was surprising, given the fact that liver is considered to be the major site of ketogenesis. While changes in both beta-hydroxybutyrate (BHB) and acetoacetate (AcAc) were statistically significant, their abundance was characterized by substantial scattering in the RCC group and few very high readings—otherwise there was major overlap between the groups (Figure S2A). Worth noting is the fact, that high ketone abundance was detected only in RCC patients and only in this group AcAc was correlated with acetone (Figure S2B). However, there was no synergistic effect when BHB and AcAc signals were combined in a multiple logistic regression model indicating that both ketones carry redundant information. Furthermore, addition of BHB and AcAc to the previously constructed multiple logistic regression model, based on lactate, NAC2, and histidine, had only minimal effect on the performance, again indicating that ketones are not a strong predictor of RCC status (data not shown). Therefore, while it seems that some changes in circulating ketones may occur in RCC, they have limited predictive power, and may be associated with secondary effects such as dietary habits and fasting duration.

Taken together, this data indicates, that quantification of serum metabolic profiles using ^1^H NMR provide sufficient amount of information for identification of RCC patient either based on multivariate PLS-DA or multiple logistic regression model.

### 2.3. Gender-Specific Differences in Control Group

**Figure 3:**
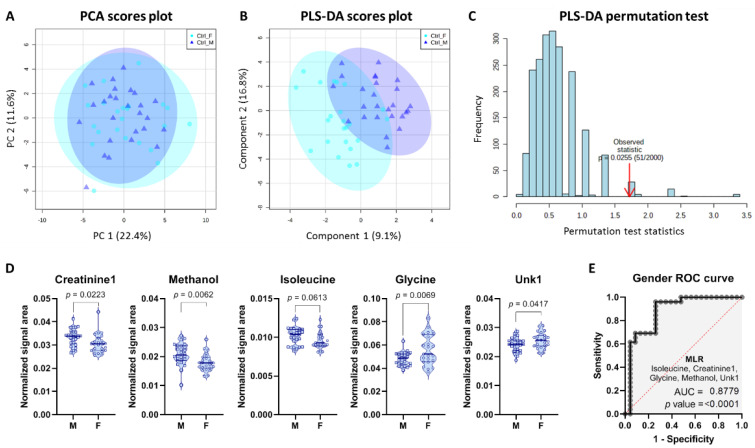
Chemometric analysis based on metabolites detected in serum of Control males and females. (**A**) PCA scores plot, (**B**) PLS-DA scores plot, and (**C**) permutation test with respect to separation distance (2000 permutations). (**D**) Five most differential metabolites selected based on their VIP score. (**E**) Combined predictive power of creatinine1, methanol, isoleucine, glycine, and Unk1 using MLR. M—Male; F—Female.

In order to investigate gender-specific differences and their potential relationship with RCC status, we next focused on the Control group alone. Control males and females overlapped in the PCA scores plot (Figure 3A) indicating a high degree of similarity between the two genders. However, by utilizing discriminant analysis we were able to partially separate the Control males from the Control females (Figure 3B). The obtained PLS-DA model was borderline statistically significant (*p* = 0.0245) and showed moderate performance in the permutation test (Figure 3C). Nevertheless, four metabolites selected based on VIP scores were statistically significant (VIP score > 1), when analyzed by means of univariate analysis, while isoleucine had *p* value of 0.06 little above statistical significance (Figure 3D). Creatinine, methanol, and isoleucine were higher, while glycine and Unk1 were lower among males (Figure 3D). Importantly, a combination of these five metabolites resulted in a good MLR model with AUC of 0.88 (Figure 3E).

### 2.4. Gender-Specific Discriminatory Potential of Serum in RCC Diagnosis

**Figure 4:**
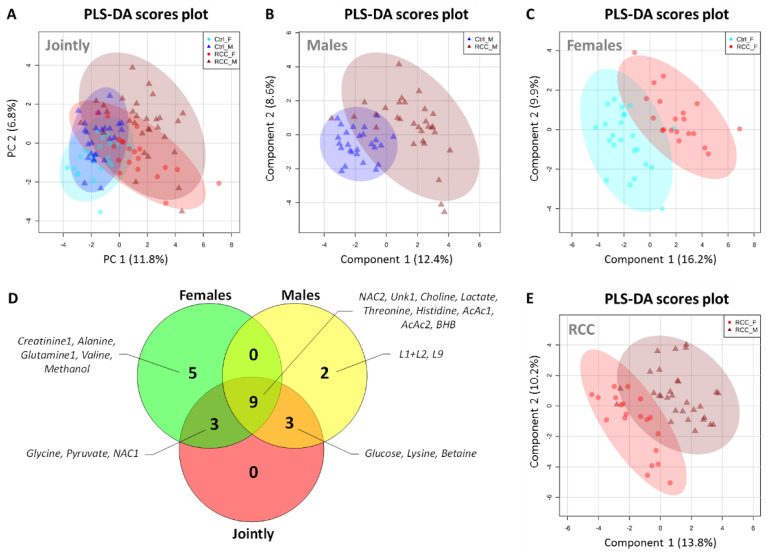
Gender-specific discriminant analysis based on metabolites detected in serum of Control and RCC patients. (**A**) PLS-DA scores plot of male and female Controls and RCC patients, (**B**) PLS-DA scores plot of male subgroup, and (**C**) PLS-DA scores plot of female subgroup. (**D**) Venn diagram highlighting the statistically significant metabolites differentiating between Controls and RCC patients selected based on VIP > 1 from PLS-DA models reported in panels Figure 4B,C and Figure 2B. (**E**) PLS-DA scores plot of the RCC patients’ subgroup discriminating based on gender. All PLS-DA models were statistically significant based on permutation test.

Given the underlying epidemiological differences in the RCC incidence ratio between males and females, we moved to explore the gender specific serum metabolic profiles. First, we repeated the chemometric analysis using all 99 samples but this time 4 groups were specified by breaking down Controls and RCC patients into male and female subjects (Figure 4A). In the PLS-DA scores plot both Control groups overlapped to a large extent, but male and female RCC patients partially separated from each other, suggesting existence of gender-specific metabolic signatures in RCC (Figure 4A). In order to further explore whether gender affect the discriminatory ability of the model, the individual PLS-DA models discriminating between RCC and Controls were built for male and female subgroups (Figure 4B,C). A similar level of separation in PLS-DA scores plot was recapitulated between Control and RCC patients when male (Figure 4B) or female (Figure 4C) subgroups were analyzed independently. Importantly, gender-specific PLS-DA models provided greater level of separation comparing to previous results where groups were analyzed jointly, suggesting existence of unique metabolic features in individual gender cohorts. 

The most differential gender specific metabolites were selected based on their VIP scores. In order to investigate the similarities between metabolites selected by the gender specific PLS-DA models we generated Venn diagram highlighting the statistically significant metabolites (Figure 4D). There were no metabolites only detected jointly, while also not being detected in at least one other comparison, indicating that there was no synergistic effect when analyzing both genders together. On the other hand, five and two metabolites were detected only in female and male specific comparisons, respectively (Figure 4D). Consistently with these differences, we were also able to obtain a good separation between male and female RCC patients, when controls were excluded from the analysis (Figure 4E).

**Figure 5:**
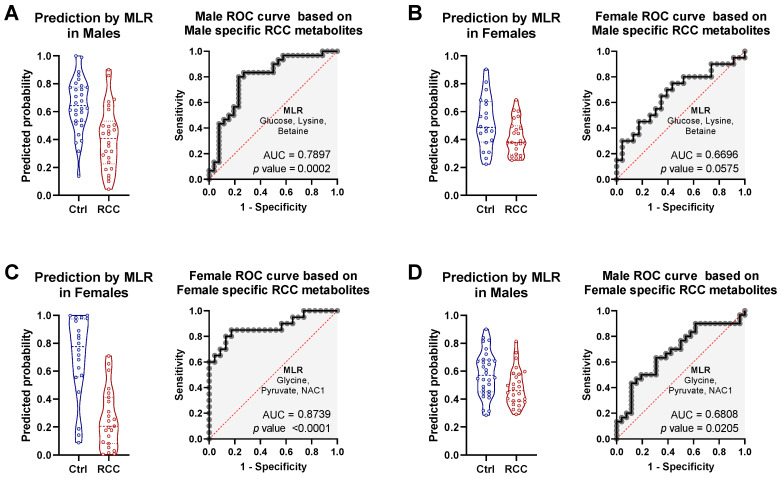
Statistical and predictive performance of MLR based on three most differential metabolites that were specific to either male or female RCC patients. (**A**,**B**) Combined predictive power of Male specific RCC metabolites (glucose, lysine, and betaine) when applied to (**A**) male cohort and (**B**) female cohort. (**C**,**D**) Combined predictive power of Female specific RCC metabolites (glycine, pyruvate, and NAC1) when applied to (**C**) female cohort and (**D**) male cohort.

Importantly, nine metabolites were detected as significantly different between RCC patients and Control by three PLS-DA models (Figure 4D). These metabolites, were considered strong overall candidates for prediction of RCC status, and mostly recapitulated the list presented in Table 1. At the same time, both female and male specific models had three unique metabolites, which were also captured by joint comparison, suggesting that these metabolites are very strongly associated with gender specific comparisons (Figure 4D), as they remain influential in the joint analysis. For males these were glucose, lysine, and betaine, while for females these were glycine, pyruvate, and NAC1. We combined these gender-specific variables into two separate MLR models and tested their performance with respect to both genders. This way we were able to evaluate how specific these MLR models are, and if they lose predictive potential when applied to the opposite gender. MLR based on male specific metabolites resulted in AUC of 0.79 and *p* = 0.0002 in the male cohort, indicating that combination of glucose, lysine, and betaine had moderate predictive power for RCC diagnosis among males (Figure 5A). Importantly, when the same set of metabolites was used in female cohort, the MLR model did not reach statistical significance (*p* = 0.0575), indicating that indeed these three metabolites were only associated with RCC status in male subjects (Figure 5B) and showed poor performance in females. The opposite pattern was observed for MLR model based on female specific metabolites, which resulted in a good predictive power (AUC = 0.87, *p* < 0.0001) for RCC diagnosis among females (Figure 5C), while in males this model was borderline significant with *p* = 0.02 and AUC of 0.68 (Figure 5D). Taken together, these data indicate existence of specific metabolic features in serum of female and male RCC patients, suggesting that different metabolic panels could potentially be used for each gender separately.

### 2.5. Discriminatory Potential of Serum Metabolie Ratios

**Figure 6:**
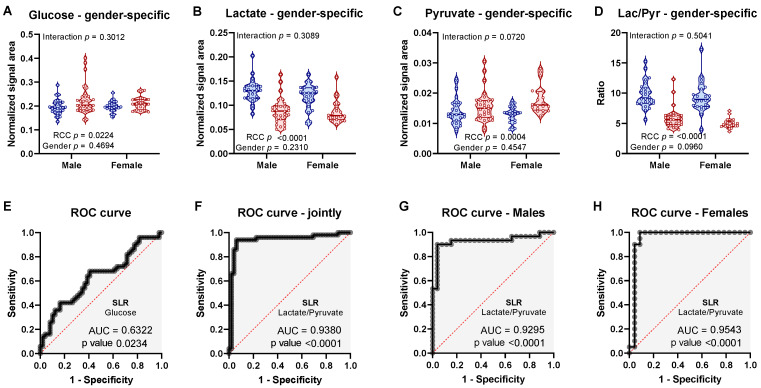
Statistical analysis by gender-specific two-way ANOVA of (**A**) glucose, (**B**) lactate, (**C**) pyruvate, and (**D**) lactate/pyruvate ratio. ROC curve from SLM for (**E**) glucose or (**F**–**H**) lactate/pyruvate ratio based on (**F**) joint analysis or individual (**G**) male and (**H**) female analysis.

Since, lactate was the strongest predictive metabolite for RCC status (Table 1) and was detected as differential by three PLS-DA models (Figure 4D), we additionally investigated its related metabolites pyruvate and glucose. Glucose, was detected to be slightly higher (Figure 6A), while lactate was decreased in RCC patients (Figure 6B) and both metabolites had no interaction with gender. Lactate and pyruvate exchange with each other at the lactate dehydrogenase (LDH) reaction and lactate/pyruvate ratio is commonly used as a surrogate of cytosolic redox in cells and tissues [30]. Although serum lactate/pyruvate ratio is not specific to any given tissues, we hypothesized, that it could reflect global whole-body changes in substrate dynamics and cellular energetics. Contrary to lactate, pyruvate levels were increased in RCC patients (Figure 6C) and its interaction with gender almost reached statistical significance (*p* = 0.07), which was consistent with pyruvate being selected by female specific PLS-DA model (Figure 4D). As a result of these opposite trends, the lactate/pyruvate ratio was a very strong measure of RCC status (Figure 6D). Unlike glucose, which had very poor predictive potential (Figure 6E), serum lactate/pyruvate ratio showed even better predictive performance than lactate itself. This was true with regard to the joint analysis (Figure 6F), as well as individual male (Figure 6G) and female (Figure 6H) analysis (in all these cases, AUC exceeded 0.9). Additionally, we investigated metabolite ratios for ketones BHB/AcAc and creatinine/creatine anticipating that they may provide synergistic effect for discrimination of RCC patients (Figure S3). RCC patients had lower BHB/AcAc ratio (Figure S3A) while creatinine/creatine ratio was not different from controls, but showed interaction with gender (Figure S3B). Taken together, these data suggest that, metabolic ratios may be a useful way for discrimination of RCC, despite their physiological meaning being challenging to interpret.

### 2.6. Gender-Sepcific Correlation Analysis

**Figure 7:**
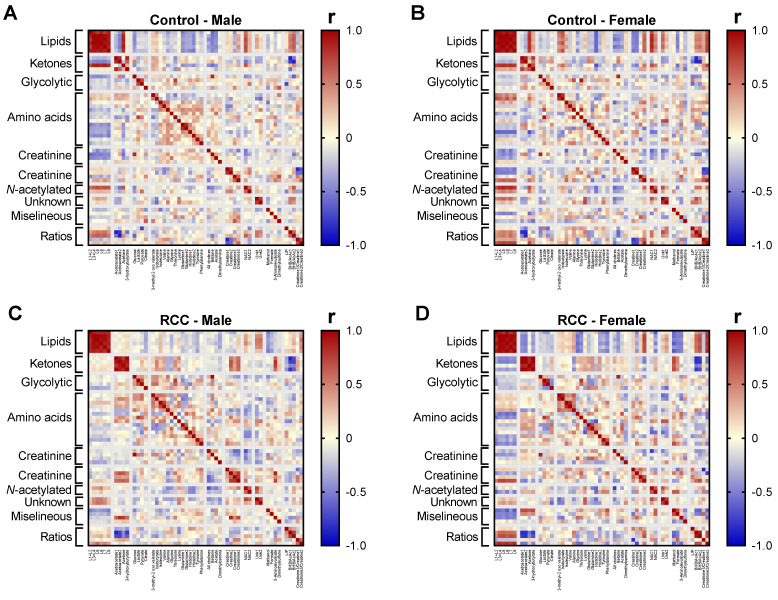
Pearson correlation map of metabolites quantified in individual gender subgroups: (**A**) Control males, (**B**) Control females, (**C**) RCC male patients, and (**D**) RCC female patients.

To further investigate the relationships between metabolites among gender-specific groups we performed correlation analysis (Figure 7). Individual heat maps reveled strong correlations within lipids across all Control (Figure 7A,B) and RCC groups (Figure 7C,D). Additionally, in both RCC male and female patients, we observed strong inner-correlation between ketones (Figure 7C,D), while this was only partially true in Controls that showed weak correlation between acetone and AcAc signals. Given the lipid origin of ketones, these two groups of metabolites are expected to be correlated with each other, but interestingly these relationships were different between Control and RCC group, again highlighting, that RCC status may somehow affect ketone dynamics in serum. Both *N*-acetylated compound signals were strongly correlated with lipids in Control male (Figure 7A) and female (Figure 7B) groups, but this correlation was very weak in male (Figure 7C) and was completely lost in female RCC patients (Figure 7D). 

Overall, the metabolite correlation map was similar between male and female Controls, consistent with the poor predictive performance of PLS-DA model generated between these groups (Figure 1B,C). Conversely, there were few distinct correlation patterns within female and male RCC patients, mostly related to the interaction between lipids, ketones, creatine/creatinine and amino acids. For example, unlike in the Controls, the creatine and creatinine signals were positively correlated in RCC groups. Despite these differences, the majority of the correlation map remained similar between genders among RCC patients.

### 2.7. Serum Metabolic Profile Is Affected by T Stage in RCC Patients

Once we established that RCC patients have differential metabolic profiles from Controls, we explored grade related changes. Although most of RCC patients analyzed in the current study were classified at the T1 stage, there were enough samples to explore stage related trends in the data. However, this analysis was conducted independent of gender due to low number of samples at higher T stage in gender specific groups. Importantly all T4 stage patients in our current study were male. 

**Figure 8:**
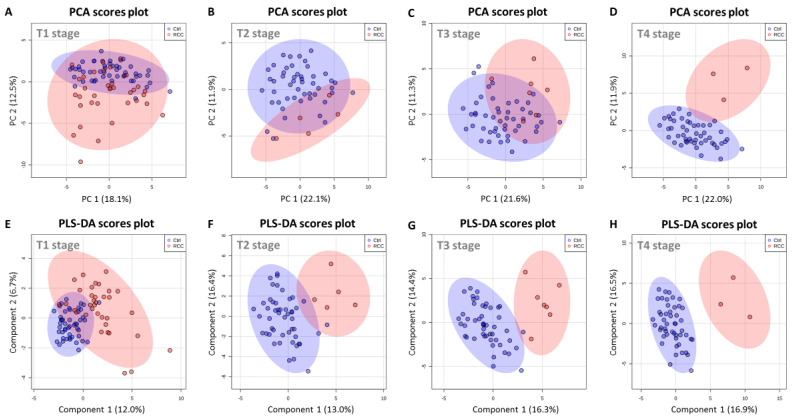
Chemometric analysis based on metabolites detected in serum of Control and RCC patients at different T stages. (**A**–**D**) PCA scores plot for Control group and RCC patients at stage (**A**) T1, (**B**) T2, (**C**) T3, and (**D**) T4. (**E**–**H**) PLS-DA scores plot for Control group and RCC patients at stage (**E**) T1, (**F**) T2, (**G**) T3, and (**H**) T4.

First, we performed series of PCA analysis where we analyzed all Controls and RCC patients at individual T stages (Figure 8). There was some degree of separation in PCA scores plot between T1 stage RCC patients and Controls, but many samples overlapped with Controls (Figure 8A). Similarly, stages T2 and T3 trended to separate in PCA (Figure 8B,C), but samples from T4 stage patients were clearly distinguishable from Control (Figure 8D). Next, we conducted subsequent PLS-DA modeling at all four T stages. Similarly, to the joint model (Figure 1B), the scores plot obtained for T1 RCC patients was characterized by some degree of overlap with Controls (Figure 8E). However, from stage T2 up to T4 (Figure 8F–H), all three PLS-DA models provided complete separation between RCC patients and Controls indicating, that T1 stage is the most challenging to discriminate. This data indicated that metabolic signatures of RCC may vary with T stage and are most clear at stage T4 consistent with the advanced degree of the disease and increased burden of the tumor on the whole-body homeostasis.

**Figure 9:**
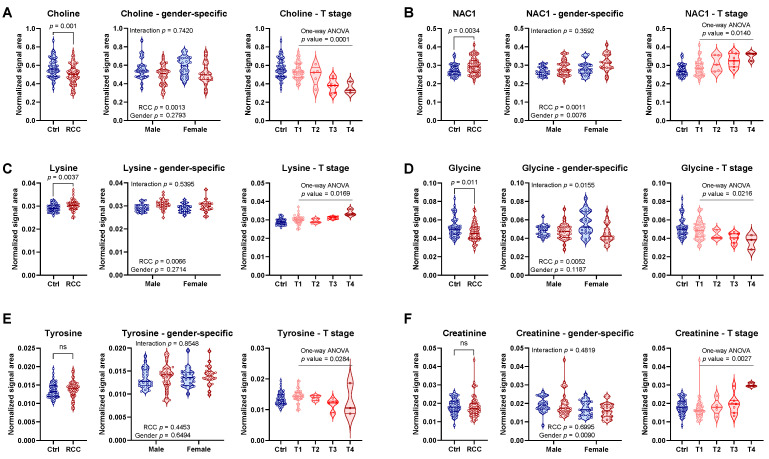
Statistical analysis of metabolites: (**left**) jointly, (**middle**) gender-specific by two-way ANOVA, and (**right**) with respect to T stage by one-way ANOVA. (**A**) Choline, (**B**) NAC1, (**C**) lysine, (**D**) glycine, (**E**) tyrosine, and (**F**) creatinine. Controls are presented in the staging panes (right) only for reference, the one-way ANOVA statistical analysis was performed using only samples from RCC patients (stages T1–T4).

In order to further explore stage related trends in the data we broke down the RCC group into subgroups determined by T stage and investigated individual metabolites for statistical significance based on the one-way ANOVA. This particular analysis was conducted excluding the Control group; however, Controls are presented in Figure 9 for reference. Although, staging analysis was conducted jointly, we additionally investigated metabolites in terms of their RCC status and gender interaction by means of two-way ANOVA in order to evaluate if gender could be a confounding factor in the T stage analysis. We assumed that metabolites without gender interaction should provide a straightforward interpretation with regard to staging when samples were analyzed jointly. 

Among the metabolites that showed significant differences based on univariate statistics regardless of stage, some of them showed additional trends when stratified by T stage. In particular choline in which downregulation was detected by the overall analysis and had no interaction with gender, showed a very significant (*p* = 0.0001) trend in relation to the T stage (Figure 9A). Conversely, NAC1, which was upregulated in joint analysis and showed no interaction with gender (although was different between males and females in both Control and RCC groups), presented a statistically significant trend dependent on T stage (Figure 9B). Lysine was also increased in RCC group regardless of gender but exhibited significant increasing trend with respect to T stage (Figure 9C). 

Interestingly, few metabolites that had poor performance in the joint analysis (or even missed threshold of significance based on VIP value or univariate statistics), exhibited strong T stage related characteristics. For example, glycine, which had *p* value of only 0.011, was also statistically significant with respect to T staging (Figure 9D). Although glycine exhibited a statistically significant interaction between RCC status and gender, it showed a very strong decreasing trend in relation to T stage, which did not seem to be affected by gender (Figure 9D). Tyrosine on the other hand, was not statistically significant when analyzed jointly or by gender specific groups, yet was associated with T stage (Figure 9E). Perhaps most striking observation was made with regard to creatinine, which was not different when RCC group was analyzed jointly, but it showed significance based on ANOVA. In particular, all three samples from patients at stage T4 showed significantly elevated levels of creatinine comparing to samples obtained from patients at lower stages or controls. Although creatinine was overall significantly higher in males than in females, this striking difference presented at stage T4 seemed to be associated with the disease status independent of gender, as these samples separated also from other male patients (Figure 9F).

Taken together these data indicate that metabolic signatures of RCC progression are present in serum and can be detected by ^1^H NMR; however, the degree with which these changes occur could be dependent on gender, as some of these metabolites were significantly different between males and females.

## 3. Discussion

In the current study, we evaluated serum metabolic profiles of RCC patients in joint and gender-specific manner. The goal of this investigation was to select differential metabolites for RCC status and to assess whether gender-specific metabolic signatures of RCC can be leveraged for a more precise diagnostic.

### 3.1. Metabolic Differences Associated with Gender and RCC

Our analysis revealed existence of moderate metabolic differences between male and female controls. Among identified differential metabolites some has previously been associated with gender and showed same trends in our study. In particular, serum creatinine is commonly reported to be higher among men based on differences in muscle mass [31,32,33,34]. At the same time, serum amino acid profiles are consistent with our finding of lower isoleucine and increased glycine in females [35]. On the other hand, serum fatty acids and various lipids (including LDL and VLDL) were previously reported to be higher among females [34,36,37], yet we were unable to detect differences in lipids among Controls in our study. Since we did not control our analysis for BMI it is possible that similarity in lipids was due to variable level of adiposity. However, in some studies that controlled for BMI, adjusting for BMI did not affect overall observed gender related differences [38], thus suggesting a potentially different nature of our observations.

Although we detected only moderate changes in metabolic profiles of Control males and females, there were substantial gender related differences among RCC patients. Similarly, to the Control group, male RCC patients had higher creatinine levels comparing to female RCC patients, indicating, that some of gender related differences are maintained despite RCC status. However, the strongest changes were associated with increased lipids among RCC females, which is consistent with typically observed trend of higher fatty acids and lipid among healthy women [34,36,37]. At the same time, male RCC patients had significantly reduced lipids comparing to male Controls, while female RCC patients had unchanged lipids compared to female Controls. Therefore, at the current state it is hard to interpret the changes in lipid signals observed in RCC females as they could be either due to common sex differences or may be associated with changes in lipid profile in male RCC patients. Confirmation of this finding by future studies will be required.

Importantly, although a number of differential metabolites was common for male and female RCC patients, there were some gender specific metabolic features associated with RCC status. A proof-of-concept MLR models based on three gender specific metabolites (glycine, pyruvate, and NAC1 for females and glucose, lysine, and betaine for males) provided good predictive power for their respective genders, and had limited utility when applied for other patients. Therefore, gender specific metabolic models could be advantageous for RCC diagnosis. 

### 3.2. Low Circulating Lactate in Relationship with Warburg Effect in RCC

Lactate, pyruvate, and their ratio as well as glucose were strongly associated with RCC status in our study. In kidney cancer, mutations in the enzymes of the TCA cycle such as succinate dehydrogenase (SDH) and fumarate hydratase (FH) lead to impairment of oxidative metabolism [39]. This suggests that RCC may exhibit classic Warburg effect, where glucose is incompletely oxidized by the tumor in the presence of oxygen and conversion of glucose to lactate is being favored. Indeed, using hyperpolarized pyruvate coupled with real-time detection of ^13^C NMR signals Keshari et al. showed that RCC cells had a significantly higher conversion rate of pyruvate to lactate compared to normal renal tubular cells [40,41]. Similarly, infusion of ^13^C glucose in ccRCC patients followed by the isotopomer analysis of metabolites extracted from tumor tissues, indicated increased flux from glucose to lactate with impairment in oxidation of glucose derived carbon in the TCA cycle, demonstrating that ccRCC is the first among human tumors that exhibit convincing shift toward glycolytic metabolism [42]. Based on these studies one could expect the circulating levels of lactate to be increased among RCC patients. However, the metabolomics data on this are contradictory. For example, higher levels of lactate were detected in RCC patients [19,21], yet at the same time lower lactate levels were associated with advanced RCC compared to low grade RCC patients without metastases [19]. Conversely, other studies reported overall decrease in blood lactate among RCC patients comparing to controls [20,22]. Consistent with latter reports, our results indicated that the decrease in serum lactate was one of the strongest predictors of RCC status regardless of gender. On the other hand, pyruvate, tends to be consistently detected in higher concentrations among RCC patients [19,25,28], which agrees with our results particularly with respect to female RCC patients. Finally, blood glucose levels are variable in RCC patients with some studies reporting it to be lower [19], unchanged [22] or increased [20]. Our study agrees with the latter studies and detected increased blood glucose in both genders, although effect was more obvious in males. 

Besides the liver, the kidney is the only organ capable of meaningful gluconeogenesis. Based on increased levels of circulating glucose in combination with decreased concentration of lactate it is tempting to suggest upregulation of gluconeogenesis in RCC patient group. However, it is unlikely that a renal tumor would produce glucose by itself. First, the genes controlling renal gluconeogenesis (G6PC, PCK1, and FBP1) were found to be the most under expressed metabolic gene set based on analysis of RNA-sequencing data of ccRCC [43] and secondly, overexpression of gluconeogenic enzyme FBP1 significantly inhibited 2D culture and xenograft tumor growth [43]. On the other hand, renal gluconeogenesis can occur under normal physiological conditions, yet is typically considered to be an important source of glucose only during acidosis and after prolonged fasting [44]. This could be important for the interpretation of serum metabolomics data, where low levels of lactate are being detected. Renal gluconeogenesis is negatively correlated with the pH, and thus is increased under acidosis [45]. Moreover, kidney can use lactate similar to liver for gluconeogenesis [44]. Lactate uptake is greater with lower pH [46] and under conditions of exogenous hyperlactatemia kidney could be responsible for up to 25–30% clearance of lactate from the blood [46]. Uptake of lactate is mostly metabolic with excretion accounting for only 10–12% of renal lactate disposal, but is not necessarily only gluconeogenic, as it was found that kidney can take up lactate and secrete portion of it as pyruvate [47,48].

**Figure 10:**
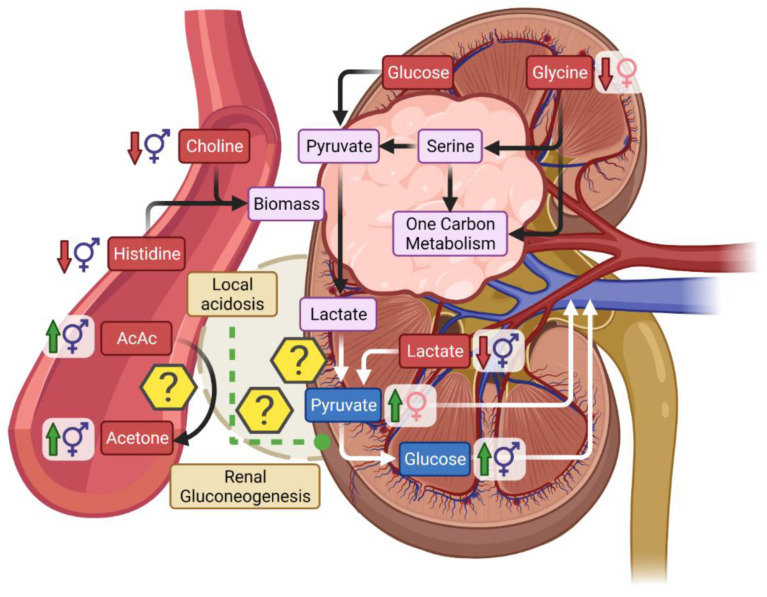
Scheme of potential metabolic changes occurring in the tumor and how tumor metabolism can affect renal metabolism. Red—arterial metabolites, violet—tumor metabolites, and blue—venous metabolites. Gender symbols refer to the primary driver of metabolic changes, for example, female increased means metabolite is increased in RCC but had significant interaction with gender which was primarily driven by females.

Production of lactate by kidney tumor could lower the local pH [49] and thus affect metabolism of the otherwise healthy rest of the kidney (Figure 10). As a result, lactate would not be secreted to circulation but rather could be converted to pyruvate, and either secreted, or used for gluconeogenesis. The net increase in circulating glucose, could be a result of tumor metabolism of other lactate precursors such as amino acids (e.g., glycine) or conversion of circulating lactate (not derived from tumor metabolism) by the same mechanism, since the local kidney metabolism already underwent a shift due to pH changes. Indeed, although rare, the case studies of RCC patient experiencing unexplained hyperglycemia exist [50,51]. Importantly, in most of these patients no major changes in hormones were detected and hyperglycemia resolved after partial nephrectomy allowing discontinuation of insulin therapy and thus suggesting that onset of hyperglycemia could have been metabolic in nature.

### 3.3. Ketones in RCC 

Ketones, BHB, and AcAc as well as acetone were all detected in greater concentration among RCC patients than in controls. Ketogenesis is generally thought to occur solely in the liver [52,53] and this would indicate that changes in ketones among RCC patients may be associated with secondary effects such as dietary habits, and fasting duration. However, the kidney also expresses 3-hydroxy-3-methylglutaryl-CoA synthase 2 (HMGCS2), the key enzyme of ketogenesis [54]. Furthermore, although kidney act as a net consumer of ketones [55], it has been reported that kidney can produce ketones in isolation [56], in vivo during starvation [57] or in diabetic nephropathy [58]. Therefore, although unlikely, it is worth mentioning that observed elevated levels of ketones in RCC could theoretically be associated with renal and not hepatic metabolism. Additionally, it is worth noting that AcAc was correlated with acetone only in RCC patients. A decrease in local pH caused by a tumor releasing lactate could contribute to elevated levels of acetone. AcAc is unstable and can spontaneously decarboxylate to acetone but this process is more rapid in lower pH [53]. Therefore, regardless of the source of AcAc, formation of acetone could be associated with the tumor itself (Figure 10). 

### 3.4. Serum Creatinine Is a Poor Marker of Early RCC 

Filtration is one of the most important roles performed by kidneys to maintain whole body homeostasis. Changes in filtration rate could affect concentration of metabolites in the blood. However, metabolites identified to be differential for RCC showed both increasing and decreasing changes, which suggest that they were not caused by only impaired renal filtration. A common although limited indicator of kidney function is serum creatinine level [32]. Creatinine is formed from creatine as a waste product of muscle metabolism and it is removed from the bloodstream by kidneys and disposed into the urine. Interestingly, neither creatinine nor creatine were changed in serum of RCC patients when analyzed jointly. However, when RCC group was broken down based on the T grade, additional insights into the dataset were obtained, while no differences in carnitine level could be observed when all RCC patients were analyzed together, we found that samples from T4 patients were characterized by significantly upregulated creatinine compared not only to the control group, but also to all other RCC patients. Although the number of subjects in each group differed, the data indicated an increasing creatinine trend with increasing T grade. Since, the rise in blood carnitine is associated with damage to nephrons it is a late marker of renal disease. Therefore, our data suggest that most RCC patients in this study maintained renal function, yet at the same time our data is consistent with the damage to kidney occurring at late stages of the disease and thus limits the applicability of serum carnitine as an early marker of RCC. 

## 4. Materials and Methods

### 4.1. Research Material Description

The study group consisted of 50 RCC patients aged 39–87 (mean age: 64.4 years), including 20 women and 30 men. All participants agreed to participate in the study by signing an appropriate form approved by the Bioethics Committee of the Wroclaw Medical University (Opinion No. KB-102/2012 of 10 April 2012). The patients diagnosed with kidney tumor were eligible for surgical treatment based on imaging examinations such as abdominal CT and MRI scans. The clinical stage was determined according to the 2012 IUAC TNM classification. The RCC patients’ characteristics are shown in Table 2. 

The control group consisted of 50 patients with no evidence of kidney tumor whom volunteered to participate in the study conducted by the Department of Ophthalmology. Blood serum was the material used for metabolomic analysis. It was collected on the day before surgery during standard laboratory tests. Blood was collected into 10 mL BD Vacutainer tubes with Becton Dickinson clot activator. After the blood clot was obtained, the serum was centrifuged at 3000× *g* for 10 min. The obtained serum was frozen at −70 °C. The frozen samples obtained from the study group and the control group were used for the metabolomic analysis using ^1^H NMR spectroscopy.

### 4.2. Sample Preparation

Prior to the metabolomics experiments, serum samples were thawed and vortexed. Subsequently, 200 μL aliquot was taken from each sample and mixed with 400 μL of saline solution (0.9% NaCl; 15% D_2_O; 3 mM TSP). After centrifugation (12,000× *g* for 10 min), a 550 μL of each sample supernatant was subsequently transferred to a 5-mm NMR tube. Samples were maintained at 4 °C prior to measurement.

### 4.3. ^1^H NMR Spectroscopy Measurements and Metabolite Assignments

All ^1^H NMR spectra were recorded at 300 K using an Avance II spectrometer (Bruker, GmbH, Bremen, Germany) operating at proton frequency of 600.58 MHz. A one-dimensional Carr–Purcell–Meiboom–Gill (CPMG) spin echo pulse sequence with water suppression was utilized (Burker library: cpmgpr1d). This allowed removal of broad spectral resonances originating from macromolecules and improved the visibility of low molecular weight metabolites. For each sample, 128 consecutive scans were collected with a 400 μs spin-echo delay, 80 loops, a 3.5 s relaxation delay, 64 K TD, and 20.01 ppm SW. The spectra were Fourier transformed with 0.3 Hz line broadening, manually phased and baseline corrected using Topspin 1.3 software (Bruker, GmbH, Bremen, Germany), and referenced to an α-glucose signal (δ = 5.225 ppm). Metabolites were identified based on previously reported assignments in serum [59,60], ChenomX metabolite library, our in-house spectral library, and statistical total correlation spectroscopy analysis.

### 4.4. Data Processing and Chemometric Data Analysis

All spectra were exported to Matlab (Matlab v. 8.3.0.532) for pre-processing. Regions affected by solvent suppression were excluded (4.32–5.15 ppm). Signals alignment was performed by the correlation of optimized warping (COW) and interval correlation shifting (icoshift) algorithms [61]. The spectra consisted of 8910 data points and were normalized using the probabilistic quotient method (PQN) where all spectra were used as reference group [62]. This allowed to overcome signal variability caused by different level of sample dilution. 

The multivariate and statistical data analysis was performed on a set of the 42 (+2 unknown) assigned metabolites. The relative concentration of metabolite measured by NMR was obtained as the sum of data points in data matrix for the no overlapping resonances (or a part of partly overlapping resonances) range. Principal component analysis (PCA) and partial least squares discrimination analysis (PLS-DA) were conducted in Metaboanalyst v5.0 using log transformed and autoscaled data. The PLS-DA models statistical significance was tested based on the permutation test with respect to separation distance (2000 permutations).

### 4.5. Statistical Analysis

Student’s t-test, analysis of variance (ANOVA), as well as simple and multiple logistic regression analysis were conducted in GraphPad Prism version 9.1.1. For univariate Student’s t-test equal variance was assumed. For logistic regression, the RCC status was set as a categorical variable Y encoding outcome, while either single or multiple metabolite abundances were set as continuous variables with main effects. One-way analysis of variance (ANOVA) was used to determine statistical significance of metabolites with respect to T stage, with α = 0.05 for statistical significance. Two-way ANOVA with interaction was used to determine statistical significance of metabolites with respect to gender and RCC status.

## Figures and Tables

**Table 1:** Differential metabolites between Controls and RCC patients.

Metabolite	VIP ^a^	Change (%) ^b^	AUC ^c^	*p* Value ^c^
Lactate	2.4590	−29.2%	0.8518	<0.0001
NAC2	2.1876	+30.7%	0.7971	<0.0001
Threonine	2.0266	−20.8%	0.7951	<0.0001
Histidine	1.7171	−16.2%	0.7322	<0.0001
Unk1	1.7071	+10.9%	0.7318	<0.0001
BHB	1.5806	+18.8%	0.7298	<0.0001
AcAc1	1.5401	+41.4%	0.7155	0.0002
AcAc2	1.4778	+46.4%	0.7049	0.0004
Pyruvate	1.3728	+21.9%	0.698	0.0007
Choline	1.3658	−12.8%	0.6869	0.0013
Lysine	1.1665	+4.2%	0.6796	0.0021
NAC1	1.1630	+8.6%	0.6563	0.0074

^a^ Variable Importance Projections (VIPs) scores obtained from PLS-DA model. ^b^ Percentage change of metabolite levels. ^c^ Area under curve (AUC) and *p* value obtained from single logistic regression for individual metabolites.

**Table 2:** Patient characteristics.

	Jointly	Male	Female
No. patients	50	30	20
Mean age	64.4	64.3	64.7
(range) Median BMI Diabetes	(32–87) - 5	(32–82) 24.8 2	(51–87) 23.6 3
Tumor stage (pT)			
pT1	35	20	15
pT2	5	2	3
pT3	7	5	2
pT4	3	3	0
RCC subtype			
Clear cell RCC	41	23	18
Papillary RCC	5	5	0
Chromophobe RCC	4	2	2
Fuhrman Grade			
1	18	10	8
2	21	14	7
3	10	6	4
4	1	0	1

## Data Availability

Original data are available from the authors on request bacause of its usage in the ongoing study.

## Supplementary Materials

The following are available online at https://www.mdpi.com/article/10.3390/metabo11110767/s1, Figure S1: Chemometric analysis based on metabolites detected in serum of 50 control and 50 RCC patients, Figure S2: Statistical analysis of ketones., Figure S3: Statistical analysis of metabolite ratios based on different resonances with respect to gender, Table S1: List of detected, assigned and quantified metabolite signals in ^1^H NMR spectra of serum.
