# Supplementary material for: Gender-Specific Metabolomics Approach to Kidney Cancer
Source: Metabolites. 2021 Nov 10;11(11):767. doi: 10.3390/metabo11110767 (PMC8624667; doi:10.3390/metabo11110767)
Supplement: Supplementary file 1 [file metabolites-11-00767-s001.zip › metabolites-1407033-supplementary.pdf]

Supplementary Data

Gender-Specific Metabolomics Approach to Kidney Cancer

Stanisław Deja 1,2,3,†,*, Adam Litarski 4,†, Karolina Anna Mielko 5, Natalia Pudełko-Malik 5, Wojciech Wojtowicz 5, Adam Zabek 5, Tomasz Szydełko 4, Piotr Młynarz 5,*

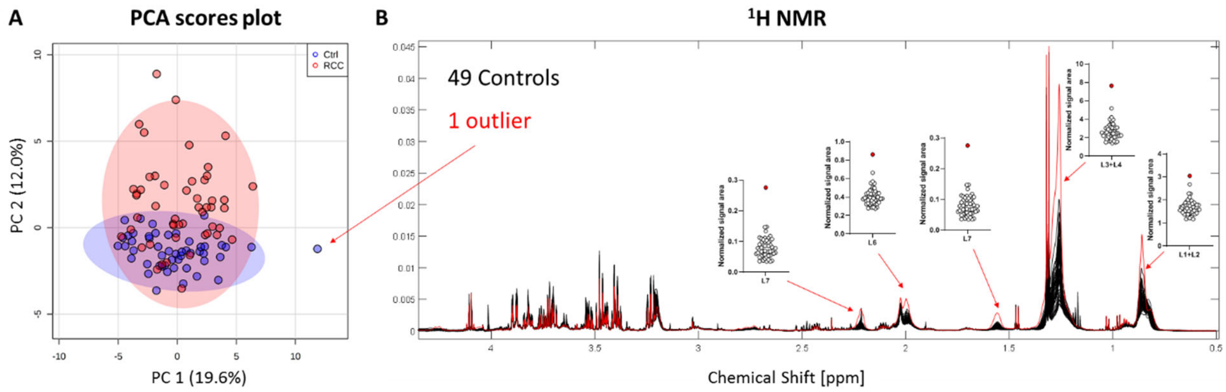

Figure S1. Chemometric analysis based on metabolites detected in serum of 50 Control and 50 RCC patients. (**A**) PCA scores plot detecting an outlier sample in Controls, (**B**) overlay of 50 Control ^{1}H NMR serum spectra. Outlier sample in red, other 49 Control samples in black. Outlier was detected based on Grubbs statistical test which is used to find an outlier in a normally distributed data set. Sample was outlier in Controls group (significance level for outlier detection was α=0.01) or the whole dataset (Control plus RCC, significance level for outlier detection was α=0.001).

Table S1. List of detected assigned and quantified metabolite signals in ^{1}H NMR spectra of serum.

Metabolite ID	Metabolite assignment	Chemical shift [ppm]	Integral Start	Integral End
		**Lipids**		
L1	Lipid LDL $C\underline{\mathbf{H}}_3$–$(CH_2)_n$–	0.830 (**b**)	0.780	0.847
L2	Lipid VLDL $C\underline{\mathbf{H}}_3$–$(CH_2)_n$–	0.860 (**b**)	0.847	0.894
L3	Lipid LDL CH_3–$(\underline{\mathbf{CH_2}})_n$–	1.248 (**b**)	1.200	1.293
L4	Lipid VLDL CH_3–$(\underline{\mathbf{CH_2}})_n$–	1.297 (**b**)	1.293	1.302
L5	Lipid VLDL –$C\underline{\mathbf{H}}_2$–$CH_2$–C=O	1.555 (**b**)	1.520	1.610
L6	Lipid –$C\underline{\mathbf{H}}_2$–CH=CH–	1.996 (**b**)	1.970	2.013
L7	Lipid –$C\underline{\mathbf{H}}_2$–C=O	2.236 (**b**)	2.220	2.243
L8	Unsaturated lipids **–**C**<u>H</u>**=C**<u>H</u>**–	5.269 (**b**)	5.232	5.345
		**Ketones**		
AcAc1 [δ2.26]	Acetoacetate –$C\underline{\mathbf{H}}_2$–C=O	2.265 **(s)**	2.261	2.269
AcAc2 [δ3.43]	Acetoacetate $C\underline{\mathbf{H}}_3$–C=O	3.431 **(s)**	3.428	3.433
Acetone	Acetone $C\underline{\mathbf{H}}_3$–C=O	2.215 **(s)**	2.214	2.218
BHB	β-hydoxybutyrate –$C\underline{\mathbf{H}}_2$–CH–	2.303 (**d**)	2.293	2.313
		**Glycolytic and related metabolites**		
Glucose	α-glucose O–C**<u>H</u>**(–OH)–CH	5.222 (**d**)	5.210	5.232
Lactate	L-lactate O=C–C**<u>H</u>**(–OH)–CH_3	4.100 (**q**)	4.090	4.112
Pyruvate	Pyruvate $C\underline{\mathbf{H}}_3$–C=O	2.358 (**s**)	2.356	2.361
Citrate	Citrate O=C–$C\underline{\mathbf{H}}_2$–C–OH	2.512 (**d**)	2.497	2.533

	**Amino acids and related metabolites**			
3-methyl-2 oxovalerate	3-methyl-2-oxovaleric acid -C**H**$_2$-CH$_3$	1.056 **(b)**	1.043	1.067
Isobutyrate	Isobutyrate C**H**$_3$-CH(C**H**$_3$)-C(=O)-	1.107 **(b)**	1.097	1.115
Isoleucine	L-isoleucine -CH(C_2H_5)-C**H**$_3$	0.998 **(d)**	0.996	1.003
Valine	L-valine -CH(CH_3)-C**H**$_3$	1.023 **(d)**	1.010	1.040
Alanine	L-alanine -CH(NH_2)-C**H**$_3$	1.461 **(d)**	1.447	1.475
Glycine	Glycine COOH-C**H**$_2$-NH_2	3.540 **(s)**	3.538	3.541
Threonine	L-threonine CH_3-C**H**(OH)-	4.145 **(m)**	4.131	4.159
Lysine	L-lysine -CH_2-(C**H**$_2$)$_3$)-CH-	1.707 **(m)**	1.670	1.750
Glutamine1	L-glutamine -C**H**$_2$-CH(NH_2)-	2.105 **(m)**	2.080	2.140
Glutamine2	L-glutamine -C(=O)-C**H**$_2$-	2.428 **(m)**	2.417	2.455
Histidine1	L-histidine -C-C**H**-N-CH-	7.025 **(b)**	7.021	7.035
Histidine2	L-histidine -N-C**H**-NH-	7.726 **(b)**	7.718	7.733
Tyrosine	L-tyrosine -C**H**-C(OH)-C**H**-CH	6.882 **(m)**	6.865	6.890
Phenylalanine	L-phenylalanine -C-(C**H**)$_5$-	7.411 **(m)**	7.394	7.430
	**Choline related**			
Choline	Choline -H(C**H**$_3$)$_3$	3.178 **(b)**	3.177	3.181
Betaine	Betaine -N(C**H**$_3$)$_3$	3.249 **(s)**	3.248	2.253
Acetate	Acetate C**H**$_3$-COOH	1.903 **(s)**	1.900	1.908
Dimethylamine	Dimethylamine C**H**$_3$-NH-C**H**$_3$	2.684 **(s)**	2.681	2.688
	**Creatinine related**			
Creatine1	Creatine -CH_2-N(-C**H**$_3$)-	3.023 **(s)**	3.021	3.025
Creatine2	Creatine COOH-C**H2**-N-	3.917 **(s)**	3.915	3.919
Creatinine1	Creatinine –N-C**H**$_3$	3.029 **(s)**	3.025	3.031
Creatinne2	Creatinine –N-C**H**$_2$-C(=O)-	4.041 **(s)**	4.038	4.045
	***N*-acetylated compounds (NAC)**			
NAC1	N-acetylated compound –NH-C(=O)-C**H**$_3$	2.024 **(s)**	2.018	2.034
NAC2	N-acetylated compound –NH-C(=O)-C**H**$_3$	2.058 **(s)**	2.055	2.060
	**Unknown metabolites (Unk)**			
Unk1	-	1.399 **(b)**	1.380	1.416
Unk2	-	1.424 **(b)**	1.416	1.432
	**Miscellaneous**			
Methanol	Methyl alcohol C**H**$_3$-OH	3.346 **(s)**	3.345	3.348
Formate	Formic acid O=C**H**-OH	8.440 **(s)**	8.439	8.443
5-Aminolevulinate	5-aminolevulonic acid -C**H**$_2$-NH_2	4.041 **(s)**	4.036	4.045
Dimethylsulfone	Dimethylsulfone C**H**$_3$-SO_2-C**H**$_3$	3.138 **(s)**	3.136	3.139

s –singlet, d-doublet, t-triplet, q- quartet, dd- doublet of doublets, m- multiplet, b -broad resonance

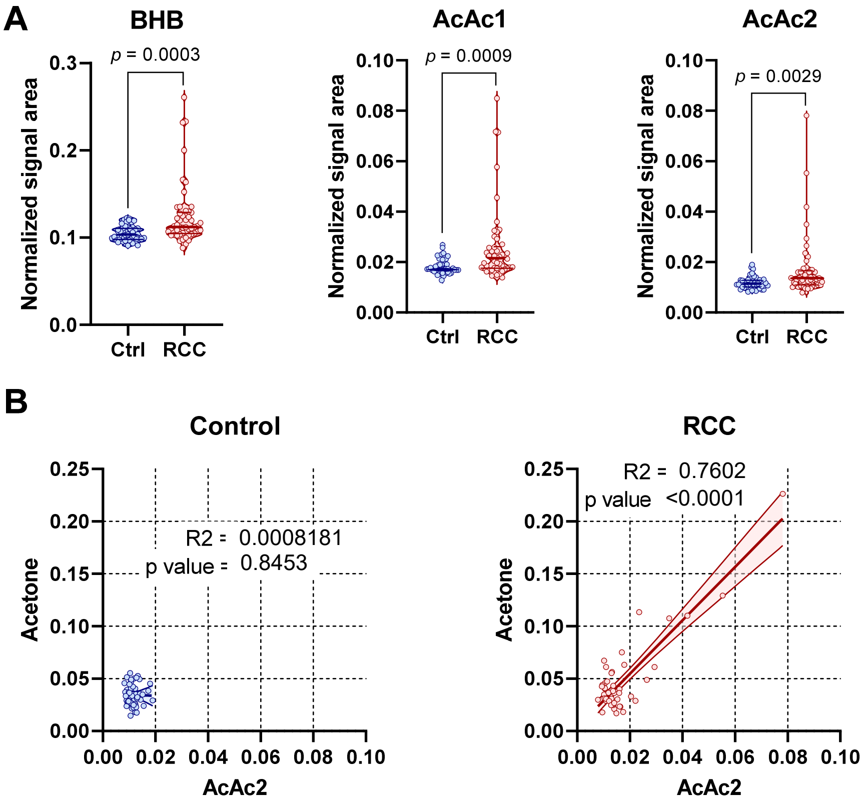

Figure S2. Statistical of ketones. (A) Univariate statistics of four ketone signals (B) relationship between AcAc2 and acetone.

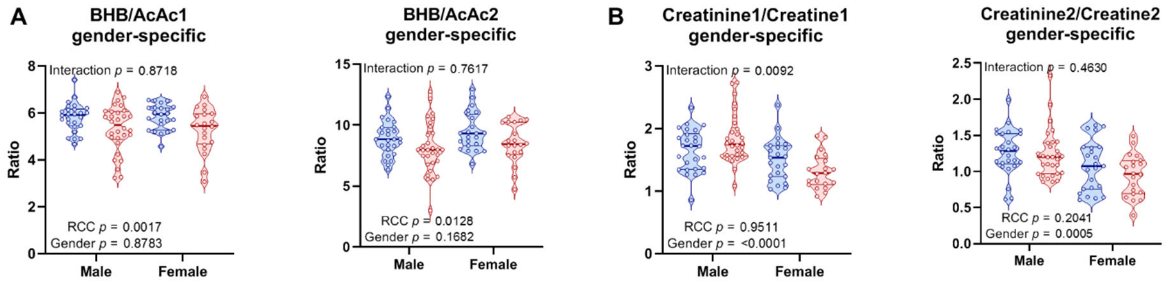

Figure S3. Statistical analysis of metabolite ratios based on different resonances with respect to gender. (**A**) ketones: BHB/AcAc and (**B**) Creatinine/Creatine.
